# Re-amputation for Intense Neuralgia in a Stump. Anæsthesia Induced by Nitrous-Oxide Gas

**Published:** 1866-02

**Authors:** 


### RE-AMPUTATION FOR INTENSE NEURALGIA IN A STUMP. ANÆSTHESIA INDUCED BY NITROUS-OXIDE GAS.

John McCollom, aged 24, of strumous diathesis, was admitted to the hospital Oct. 12, 1865. He had been serving in the Army of the Potomac. On the 28th of September, 1864, his left foot was carried away by a piece of shell, amputation was performed a few hours afterward, in the inferior third of the leg by antero-posterior flaps. Union progressed favorably for three weeks, although, even during that time, there was an unusual amount of tenderness, in the stump, but more especially in the course of the anterior tibial and peroneal nerves. Over the position of the end of the last nerve a hard nodule can be readily felt through the skin, intensely sensitive to pressure, and evidently involved in the cicatrix. Several small nodules can be felt in other parts of the stump. The skin is adherent to the end of the bones and the whole stump has the feeling of hardness, and immediately over the peroneal nerve at its termination, the skin is discolored and has frequently been the seat of ulcerations. The tissues above the stump are healthy, and his general health is good. The nodules are bulbous enlargements of the nerves which are disorganized, and the hardness is due to a deposit of fibrous matter. The only operation that will give him relief consists in a re-amputation higher up, going beyond the diseased nerves. Excisions of these tumors would not be of benefit here, as probably all the nerves are involved.

I use in this case as an anæsthetic, the protoxide of nitrogen, which consists of one equivalent of oxygen and one of nitrogen. The gas is carried in various sized gutta-percha bags, having a flexible tube and a hard rubber mouth-piece, with a stop-cock attached, which is placed against the teeth, the mouth being held open by a small wedge. The lips are drawn over the mouth-piece and, the nose being closed, the patient is directed to take a full breath. From twenty to thirty seconds is quite sufficient to induce total anæsthesia.

The patient was put under the influence of this agent in about fifteen seconds, and the amputation performed by making lateral skin flaps and a circular of the muscles, without the slighest pain or unpleasant effect. The operation lasted two minutes.

*Dissection of the Stump.* Oct. 25, 1865. Dr. Pepper reports that all the tissues in the lower part of the stump were involved in a dense cartilaginous cicatrix. There was marked enlargement of all the nerves, especially of the posterior tibial, which was fully as large as an ordinary sciatic, hard and coarse, and terminating by a fibrous cord with a hard nodule developed about an inch above its insertion into the cicatrix. Both the anterior tibial and peroneal nerves terminated in flattened nodules, directly involved in dense fibrous tissues. The vessels were patulous to within a short distance of the end of the stump.

*Microscopic Appearances.* In the anterior tibial and peroneal nerves, neurilemma was very much thickened. The nerve-tubes tortuous with deficiency of the white matter. Numerous small oil globules along the fibres. In the posterior tibial, below the point of second amputation, there was no trace of true nervous structure, the coarse fibres of which the nerves consisted being entirely composed of many bands of fibrous tissues.

